# Vets, Meds, and Zoonotic Threats

**DOI:** 10.3201/eid1004.030805

**Published:** 2004-04

**Authors:** Silvio Pitlik

**Affiliations:** * Rabin Medical Center-Beilison Campus, Petah Tikva, Israel

**Keywords:** emerging zoonoses conference

## Abstract

The fourth international conference on emerging zoonoses (September 18–21, Ames, Iowa, USA) brought together 180 scientists and healthcare specialists from 18 countries working to control diseases transmitted from animals to humans. The meeting took place under the auspices of the Center of Food Security and Public Health, USA, and the Institute for International Cooperation in Animal Biologics (a collaborating center of the World Animal Health Organisation [OIE]).

A multidisciplinary and global approach shed new light on both old and new zoonoses. For example, brucellosis topics covered a wide range of material, from economic aspects of control in Mongolia to characterization of *Brucella* isolates from feral swine in coastal South Carolina. Another presentation concerned the increasingly appreciated role of wildlife in the dynamic epidemiology of other zoonotic infections, such as tuberculosis. Scientists also explored the intricate routes prions follow between wildlife and domestic animals; between sheep, cattle, and humans; and between the tongue and brain of infected animals.

Since most agents of bioterrorism potential are zoonotic, a full session was dedicated to bioterrorism and biodefense. It included a global view, a report on national preparedness by Israeli hospitals, and examples of research that may eventually help experts coping with bioterrorism but would also unfortunately be accessible to persons with malicious intent.

Innovative methods for preventing spread of foodborne pathogens were presented, including the use of fluorescence spectroscopy to detect fecal contamination on animal carcasses or the use of vaccination to reduce transmission of zoonotic pathogens and drug-resistant nonpathogens through the food chain to humans. In the field of xenotransplantation, key components of a source-animal production facility were described. The feasibility of breeding pigs free of designated pathogens offers hope for wide use of xenotransplantation in the near future.

Participants also discussed current trends and challenges of protozoan parasitic zoonoses, including cryptosporidiosis, toxoplasmosis, African and Latin American trypanosomiasis, and leishmaniasis. Controversial zoonotic viruses were given an important place in the conference. These included hepatitis E virus, with similar strains causing liver disease in swine and humans; Borna disease virus, causing neurologic disease in various species of animals as well as, debatably, psychiatric disorders in humans; and the recently discovered severe acute respiratory syndrome–associated coronavirus and its yet-undefined animal reservoir. The recent mapping of the genome of *Mycobacterium avium* subspecies paratuberculosis, the etiologic agent of Jhone’s disease in cows, brought some hope in solving the long-lasting dispute on its role in the pathogenesis of Crohn’s disease in humans.

The value of using a global, multidisciplinary approach was highlighted in studies on the flow of genes among avian, swine, and other influenza viruses and on the ongoing intercontinental spread of arboviruses, exemplified by the evolving epizootic of equine West Nile encephalitis in the United States. Several papers dealt with the epidemiology of Nipah, Ebola, monkeypox, rabies, and Hantaan viruses.

A series of presentations demonstrated how genomic fingerprinting and other sophisticated molecular biology techniques allow exceptionally fast development in understanding the epidemiology and pathogenesis of many zoonotic infections, such as those caused by *Escherichia coli* O157:H7 or by species of *Anaplasma*, *Bartonella*, *Borrelia*, *Campylobacter*, *Coxiella*, *Francisella*, *Pasteurella*, and *Salmonella*.

The “one-track” meeting, by avoiding parallel and superspecialized sessions, gave an opportunity for fruitful and inspiring interactions among experts from multiple disciplines with a shared goal of mitigating human disease from emerging infections. More details on the meeting can be viewed online (available at: http://www.zoonoses2003.com).

